# Using X-ray Diffraction Techniques for Biomimetic Drug Development, Formulation, and Polymorphic Characterization

**DOI:** 10.3390/biomimetics6010001

**Published:** 2020-12-30

**Authors:** Israel Rodríguez, Ritika Gautam, Arthur D. Tinoco

**Affiliations:** 1 Department of Chemistry, University of Puerto Rico Río Piedras, San Juan, PR 00925, USA; 2 Department of Chemistry, Indian Institute of Technology Kanpur, Kanpur 208016, India

**Keywords:** drug products, active pharmaceutical ingredient, amorphous solid dispersions, excipients, X-ray crystallography, pair distribution function

## Abstract

Drug development is a decades-long, multibillion dollar investment that often limits itself. To decrease the time to drug approval, efforts are focused on drug targets and drug formulation for optimal biocompatibility and efficacy. X-ray structural characterization approaches have catalyzed the drug discovery and design process. Single crystal X-ray diffraction (SCXRD) reveals important structural details and molecular interactions for the manifestation of a disease or for therapeutic effect. Powder X-ray diffraction (PXRD) has provided a method to determine the different phases, purity, and stability of biological drug compounds that possess crystallinity. Recently, synchrotron sources have enabled wider access to the study of noncrystalline or amorphous solids. One valuable technique employed to determine atomic arrangements and local atom ordering of amorphous materials is the pair distribution function (PDF). PDF has been used in the study of amorphous solid dispersions (ASDs). ASDs are made up of an active pharmaceutical ingredient (API) within a drug dispersed at the molecular level in an amorphous polymeric carrier. This information is vital for appropriate formulation of a drug for stability, administration, and efficacy purposes. Natural or biomimetic products are often used as the API or the formulation agent. This review profiles the deep insights that X-ray structural techniques and associated analytical methods can offer in the development of a drug.

## 1. Introduction

The process of translating a compound of therapeutic potential from the benchtop to the drug market is a rigorous one involving many hurdles in terms of time, money, and human resources. A single compound can take as many as 20 years to become an approved drug and cost 2–3 billion of dollars in research and development [1]. To help facilitate this process and minimize the time and effort required for clinical trials and safety clearance, the research landscape for drug discovery has shifted from a focus on phenotypic discovery or empirical examination of drug candidates to a focus on biological targets [2]. By understanding the molecular mechanisms for the onset and progression of a disease, druggable targets can be discovered. Aiding in this directive is the consideration of the physico-chemical properties required for optimal use of a drug by humans including solubility and biocompatibility. Many potent active pharmaceutical ingredients (APIs) are poorly soluble and/or poorly permeable. The formulation is therefore key for the appropriate administration of the APIs to maximize their absorption into the body and their efficacy.

Looking at nature for inspiration in drug design and formulation can yield invaluable insight to optimize the potency, specificity, and storage stability of the drug products. Nature exhibits an extensive array of systems with varied function and utility, organized from the atomic level to the macro level to undergo viable life processes. Thereby, scientists find utilization of nature’s design as the most reliable and sustainable resource for the development of high performing technologies, tools, devices, materials and systems. This has, in turn, resulted in the improvement of instrumentation and methodologies that decrease the limits of detection to uncover greater details about biological processes and bioactive molecules. These details elucidate biomimetic products that can serve as APIs and/or the formulation agents known as excipients to improve drug development and administration especially given the wealth of knowledge that can be obtained regarding their bioactivity, toxicity, and metabolic fate. To this end, X-ray structural characterization techniques including single crystal X-ray diffraction (SCXRD) and powder X-ray diffraction (PXRD) using the pair distribution function (PDF), coupled with other complementary techniques have greatly contributed to biomimetic drug design and long-term assessment of drug stability. This review will demonstrate how SCXRD not only provides structural characterization but also mechanistic insights into drug targets and critical interactions within small compounds and between small compounds and macromolecules that can generate new drugs and drug candidates. It also examines how the pair distribution function has enabled PXRD to become a powerful tool to assess quality control in drug formulation and long-term shelf-life stability.

## 2. Significance of X-ray Crystallography in Drug Discovery

SCXRD has historically been the most sought-after technique for the determination of several structural features in drug molecules and has played a major role in structure-based drug design (SBDD), drug-receptor interactions, and the development of target biomolecule-ligand complexes. The first hexagonal bipyramidal structure of pepsin was prepared by Dr. Philpot in the lab of Dr. Theodor Svedberg in Uppsala and studied by Dr. Bernal, also known as Sage in the early 1930s during his position at Cambridge [3,4]. About 28 years later, Dr. John Kendrew (1958) and Dr. Max Perutz (1960) were awarded the Nobel Prize in Chemistry (1962) for determining the crystal structure of myoglobin and hemoglobin, respectively [5]. Soon after in 1964, Dr. Dorothy Crowfoot Hodgkin was awarded the Nobel Prize in Chemistry for the determination of the structure of a series of significant biochemical substances, including cholesterol, penicillin, vitamin B12, and insulin [6,7]. In recent years, there has been an exponential increase in protein-based biomacromolecular models. The popularity of the Protein Data Bank (PDB), which was formed in 1971, can be seen by a steep rise in the data submitted to it over the years, which has reached over 100,000 deposits, largely coming from X-ray crystallographic studies [8]. For scientists working in the field of drug discovery, the three-dimensional structure of proteins and their active sites play a significant role in its progression.

### 2.1. Relevance of the Structural Information of Molecules in Drug Discovery

The geometric features that play significant roles in the properties of biologically relevant molecules and ions include chirality and absolute configuration. Knowing the accurate geometry of the small molecule that can interact with the active site of a target molecule and thereby act as an activator or inhibitor, allows scientists to design drugs with therapeutic potentials.

The primary use of the structure determination of proteins and interacting drug complexes is to foster a chemical hypothesis for the binding in order to estimate drug affinity for the target protein and recommend necessary chemical amendments. Protein-drug interactions are usually steered by different kinds of intermolecular and intramolecular forces, including hydrogen bonding and the hydrophobic and hydrophilic effect of the multi-dimensional scaffold of the protein with the drug in its active binding site. This information is largely used by medicinal chemists and chemical biologists to design and synthesize a cohort of drug variants with altered inhibitory and activation activity. Thus, in the field of drug discovery, the development of novel chemical entities with high selectivity and specificity towards the therapeutic targets is of utmost importance for rational drug design. Understanding of the structural information of APIs is a necessity to qualify as a new medicine.

### 2.2. Significant Drug Properties Revealed by X-ray Crystallographic Studies

**Figure 1:**
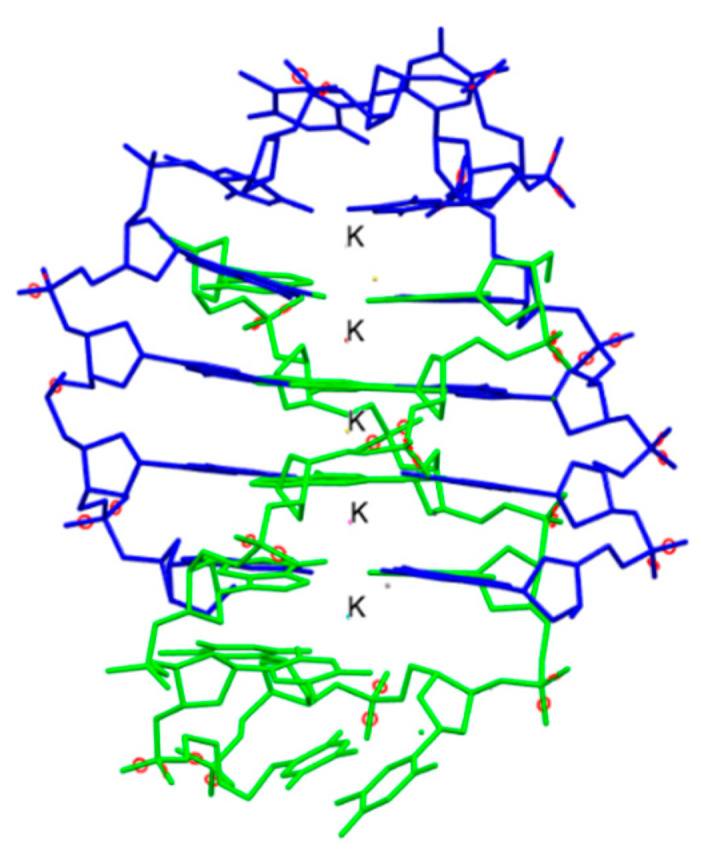
The crystal structure of the bimolecular quadruplex formed by the *Oxytricha nova* telomeric sequence d(G_4_T_4_G_4_). Potassium ions are shown with label K. Hydrogen atoms are omitted for clarity. PDB code: 1JPQ. Ref. [9].

**Figure 2:**
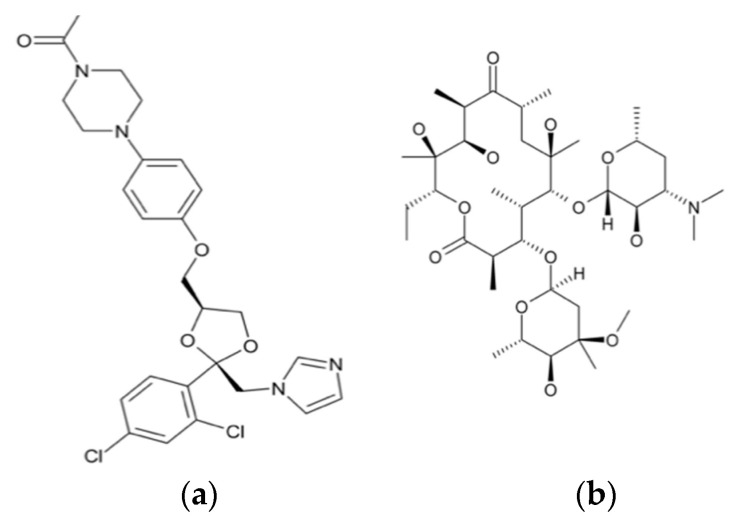
Chemical structures of ketoconazole (**a**) and erythromycin (**b**).

One of the major breakthroughs in the field of X-ray crystallography was the discovery of G-quadruplexes (Figure 1). They are higher order deoxyribonucleic acid (DNA) and ribonucleic acid (RNA) whose structures are not displayed by any other type of previously known DNA. The high-resolution X-ray crystallographic structure along with the biological understanding is significant to discover drugs that target DNA and RNA quadruplexes from specific genes [9]. Another major contribution came in 2008 with the crystal structure determination of human cytochrome P450 (CYP)3A4 in complexation with two thoroughly studied drugs: ketoconazole and erythromycin (Figure 2) [10]. Because CYP3A4 impacts the metabolism of 50% of drugs in the market, the crystal structure of the CYP3A4-ligand interaction provided crucial information regarding its binding stoichiometry (2:1 ketoconazole:CYP3A4 and multiple binding modes in the case of erythromycin) and two distinct open conformations in the presence of ketoconazole and erythromycin, and the associated kinetics of the binding process. Early in 2015, a valuable resource to direct antibacterial drug discovery was established with the revelation of the high-resolution three-dimensional X-ray structures of symbolic members of all enzymes in the type II fatty acid pathway [11]. These structures helped us to understand ligand recognition, selectivity and specificity towards the length of the chain, and the catalytic activity. Similarly, a series of X-ray crystallographic structure analysis of metalloenzyme carbonic anhydrase with the CO_2_ ligand had pharmacologic applications in the field of anti-fungal, anti-bacterial, anti-glaucoma, anti-convulsant, and anti-cancer agents [12].

**Figure 3:**
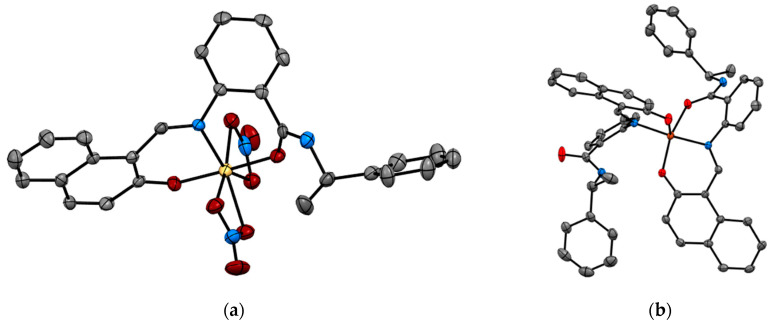
Metal complexes of sirtuin inhibitor, sirtinol with Fe(III) and Cu(II) (**a**) [Fe^III^(sirtinol-H)(NO_3_)_2_] (CCDC 1036424) and (**b**) Cu^II^(sirtinol−H)_2_ (CCDC 1402316). Hydrogen atoms are omitted for clarity. Ref. [13,14].

Recent studies by Tomat et al. explored the coordination and redox chemistry of sirtinol, a known inhibitor of the sirtuin protein. The sirtuin protein is a nicotinamide adenine dinucleotide (NAD^+^) dependent histone deacetylase. Sirtinol was long believed to solely act as an inhibitor of the sirtuin protein but recent X-ray structure determination of sirtinol in the presence of metal ions such as Fe(III) and Cu(II) indicates that sirtinol, along with inhibiting sirtuin, can coordinate biologically relevant metal ions [13,14]. This establishes that sirtinol’s biological profile is not simply a drug like interaction with the target protein and to act as an inhibitor but, interestingly, a composite of several other interactions. The single crystal structure analysis of [Fe^III^(sirtinol-H)(NO_3_)_2_] revealed the iron center is bound by monoanionic sirtinol in a tridentate coordination mode and by two nitrato ligands (Figure 3). In the case of Cu^II^(sirtinol−H)_2_, each copper center is bound to two monoanionic sirtinol ligands. This work lays the foundation for further studies to examine the difference between free sirtinol interaction with the target protein sirtuin versus transition metal coordinated sirtinol.

### 2.3. X-ray Crystallographic Structural Insights Elucidate Promising Biomimetic Drug Candidates

**Figure 4:**
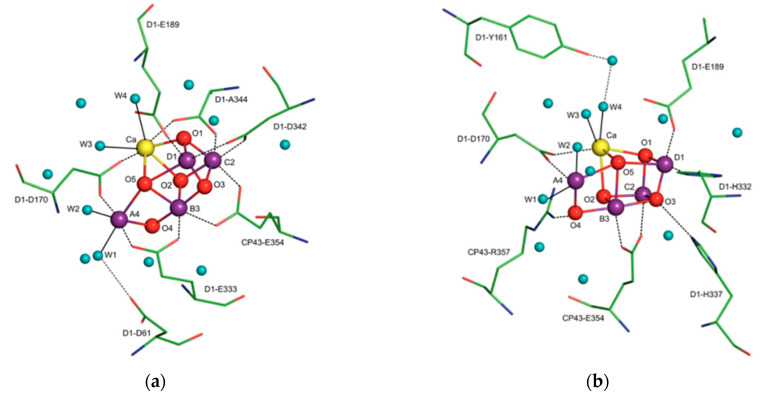
The X-ray crystal structure of the water-oxidizing complex of photosystem II. For clarity of presentation only selected amino acids are shown in views (**a**,**b**). Blue spheres, water molecules; magenta spheres, manganese ions; red spheres, μ-oxo bridges; yellow sphere, calcium. Reprinted from Biochimica et biophysica acta, 1827, Cox, N.; Messinger, J. Reflections on substrate water and dioxygen formation. 1020–1030, Copyright (2013), with permission from Elsevier.

X-ray crystallographic structures can reveal atomic and molecular details about how the natural world works. The structural components of bioactive molecules can be useful for developing technologies and aberrant processes and can help in understanding the interactions that lead to the onset of a disease. These structures have contributed enormously to the growth of the biomimetic field in which small molecules are constructed to acquire deeper mechanistic insight. A significant contribution of X-ray crystallography in the biomimetics field came with the determination of a high-resolution structure (3.8 to 2.9 Å) of photosystem II [15]. Photosystem II consists of 20 subunits with a molar mass of 350 kDa. It is the hub for the water oxidation process during photosynthesis. The structure allows us to understand how it facilitates this process and how metals play a significant role in bioenergetics. The active site consists of a Mn_4_CaO_5_ cluster (Figure 4) where the substrate (a water molecule) binds and undergoes splitting [15,16]. From a structural standpoint, the active site consists of five bridged oxo atoms that link five metal atoms and four water molecules that are coordinated to the Mn_4_CaO_5_ cluster. This structural insight inspired the development of several biomimetic compounds that have helped to measure the energy for water splitting and for the formation of molecular oxygen and have reflected on varied oxidation states of metals in different biological settings for water splitting [15,16,17,18,19]. They have also been useful for emerging applications of artificial photosynthesis [15,16,17,18,19].

**Figure 5:**
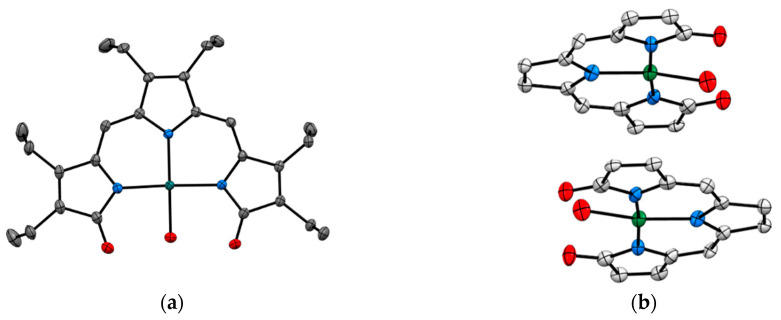
Crystal Structure of tripyrrindione transition metal complexes. (**a**) Top view of [Pd(TD1•)(H_2_O)] (CCDC 1400990) and (**b**) Stacked view of [Cu(TD1•)(H_2_O)] (CCDC 1452414). Hydrogen atoms are omitted for clarity. Ref. [20,21].

SCXRD also assists in enriching the structural chemistry of biologically relevant ligands in coordination compounds. Tripyrrindione is an analogue of the naturally occurring urinary pigment, uroerythrine. The structural characterization of tripyrrindione and its Pd(II), Cu(II), and Zn(II) complexes in different redox states were thoroughly studied by Tomat et al. (Figure 5) [20,21]. The X-ray structure determination establishes (a) stabilization of unpaired electrons that participates in π−π interactions and is vital for several electron-transfer biological processes (b) planar geometry with three pyrrolic nitrogens bound to the metal center and the fourth coordination sphere occupied by an aqua ligand and (c) dimerization in the solid state. The tripyrrindione ligand in coordination with the metal ions provides a robust platform for ligand-based redox chemistry.

X-ray crystallographic structures of metal coordination within proteins can provide valuable knowledge regarding manipulating coordination chemistry to perturb metal regulation within the human body for medicinal purposes. Such insight has been applied to the development of a recent anticancer drug design strategy. Cancer is the second main global cause of death, claiming nearly 10 million lives in 2018 according to the International Agency for Research on Cancer (IARC). Today the most commonly used treatments are the traditional approaches consisting of surgery, radiation, and the platinum(II)-based drugs. These treatments have major limitations, typically requiring early detection and, even then, having low success rates. Chemotherapeutics suffer from a lack of specificity resulting in off-target toxicity and acquired resistance. The development of the omics field, technologies used to explore biological ions and molecules of organisms, has enormously transformed cancer studies by providing deeper insight into the tumor environment at the molecular level and revealing pathways that lead to the origin of specific cancer types [22,23,24]. This work has led to the identification of new druggable targets and of ambitious treatment strategies such as immunotherapeutics [22,23,24]. In this vein, CRISPR genetic editing technology, which has resulted in the 2020 Nobel Prize in Chemistry being awarded to Dr. Emmanuelle Charpentier and Dr. Jennifer A. Doudna for its development, may prove crucial in advancing the benefits of immunotherapy. A phase 1 clinical trial was completed in the U.S. to evaluate the safety and feasibility of CRISPR-Cas9 gene editing in three patients with advanced cancer and the treatment was found to be well-tolerated [25]. While there are promising treatment strategies underway, many are in their early stages. There is a tremendous cost of time and effort, as previously stated, for a strategy to reach clinical application. Several of these strategies are likely to be too costly and thus limited in their general use. Another approach developed by the Tinoco laboratory has also arisen from omics-research and was inspired by a proposal made by Dr. Robert A. Weinberg, a leader in the oncology field, that a landmark drug will be one that attacks the pathways that keep cancer cells alive via a multi-strategic mechanism [26]. This approach targets decreasing the bioavailability of iron (Fe) to cancer cells due to the central role that Fe plays in cell development and proliferation. Cancer cells have a higher requirement for Fe than healthy cells in order to meet their greater metabolic demand [27]. As such cancer cells overexpress the transferrin receptor, a membrane bound protein, to bind Fe(III) from serum transferrin and internalize it via endocytosis [28,29,30,31]. They also increase Fe retention by shutting down the export of membrane transport proteins. The net effect is an increased labile iron pool (LIP) that is readily accessible for functionalization. Cancer cells overexpress the cytosolic Fe-dependent enzyme ribonucleotide reductase (RNR) to take advantage of this elevated pool. RNR is the only protein to produce deoxyribonucleotide diphosphates (dNDP) [32,33,34], the monomeric building blocks for DNA synthesis and also DNA repair [35]. Blocking access to the intracellular LIP would not only inhibit key enzymes that sustain cell viability but would effectively shut down cell replication.

**Figure 6:**
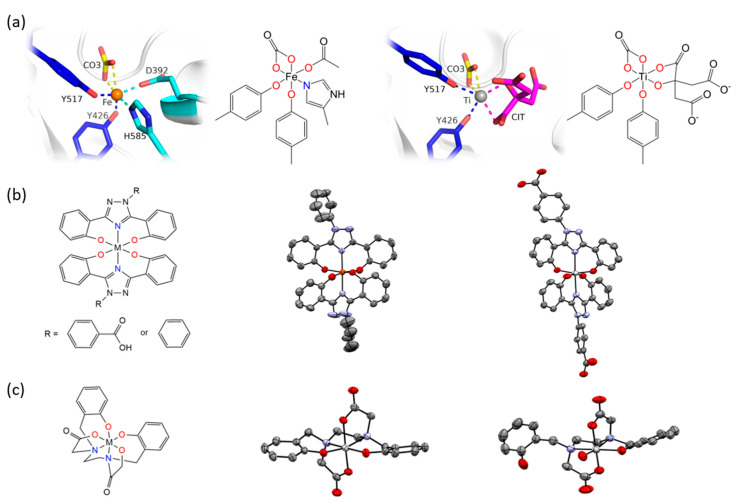
X-ray structures of Fe(III) and Ti(IV) coordination by serum transferrin (sTf) and by the small molecule chemical transferrin mimetic ligands. (**a**) The X-ray structures and chemical structures of the Fe(III) coordination modality (PDB 3QYT; left) and Ti(IV) coordination modality (PDB 5DYH; right) in the metal binding site of sTf. (**b**) The molecular representation (left) of the metal bisdeferasirox and metal bisdeferasirox analogue compounds demonstrating the meridional coordination of the ligands. The X-ray structure of [Fe(3,5-bis(2-hydroxylatephenyl)-1-phenyl-1,2,4-triazole)_2_]^−^ (middle; CCDC 237449). Ref. [55]. The cation was omitted for clarity. The X-ray structure of [Ti(Deferasirox)_2_] (right; CCDC 2016873). Ref. [57]. The hydrogen atoms were omitted for clarity. (**c**) The molecular representation (left) of the metal HBED compounds in which HBED serves as a hexadentate ligand. The X-ray structure of Ti(HBED) in which HBED coordinates the metal in hexadentate modality (middle; CCDC 645069). Ref. [59]. The hydrogen atoms were omitted for clarity. This structure is comparable to the X-ray structure of [Fe(HBED)]^−^ (CCDC 1194788; Ref. [60]). The X-ray structure of [TiO(H^+^-HBED)]^−^ in which partial metal hydrolysis resulted in the HBED ligand coordinating the metal in pentadentate modality (right; CCDC 846178). The cation was omitted for clarity. Ref. [61].

The Tinoco laboratory has devised a bioinspired dual approach to suppress Fe bioavailability within cancer cells. This approach consists of combining iron chelators with titanium(IV), a metal ion that shares many chemical properties with Fe(III) and which depending on its coordination chemistry can display potent anticancer properties [36,37,38,39,40,41,42,43,44,45,46,47]. Previously, Fe chelators that were designed for the treatment of iron overload diseases and that operate either extracellularly or intracellularly have been investigated for anticancer therapies [48]. This approach has proven extremely promising, with several repurposed chelators currently in anticancer clinical trials [49]. The choice of Fe chelators and Ti(IV) in the Tinoco laboratory approach was not trivial. Ti(IV) was judiciously selected because it is one of the few nonferric metal ions that is known to be bound endogenously in the human body by the 80 kDa Fe(III) blood transport glycoprotein serum transferrin (sTf) [41,50,51,52]. This bilobal protein, which can carry two Fe(III) ions, one in each of its lobes, is primarily responsible for Fe(III) delivery into all cells via the transferrin receptor endocytosis process. Ti(IV)-bound sTf at high concentrations (exceeding physiologically relevant levels) has been shown to block cellular uptake of Fe(III)-bound sTf [53]. This capacity of Ti(IV) to perturb Fe(III) availability to cells could be exploited by manipulating the coordination chemistry of these related metal ions to weaponize Ti(IV). To this end, the crystal structure of Ti(IV)-bound sTf was sought to understand the coordination details of how this protein stabilizes a notoriously hydrolysis prone metal and to decipher key differences in how this protein binds Fe(III) versus Ti(IV). Interestingly, the crystal structure of a physiologically relevant form of Ti(IV)-sTf demonstrated that Ti(IV) is bound in a similar but not identical modality as Fe(III) (Figure 6a). The canonical Fe(III) coordination consists of the metal ion bound to four amino acids (two tyrosines, one histidine, and one aspartic acid) and a synergistic carbonate anion [30]. Ti(IV) binds only to the two tyrosines and the carbonate. In place of the histidine and aspartic acid is a bidentate citrate anion, which serves as an additional synergistic anion [50]. As such, the Ti(IV) coordination is classified as noncanonical [52]. The citrate small molecule and sTf are believed to closely interact to regulate Ti(IV) bioactivity and minimize its toxicity but the details of this interaction is outside the scope of this review [43,50,51,52]. This difference in Ti(IV) and Fe(III) coordination by sTf is attributed to the higher Lewis acidity of Ti(IV) and informs on a template for iron chelators appropriate for the anticancer drug design strategy. Iron chelators that mimic Fe(III) coordination by sTf, termed chemical transferrin mimetics (cTfm), were selected for this study. The driving force for this selection was that these chelators have the capacity to stably bind Ti(IV) but have a higher preference for Fe(III) binding. N,N′-di(o-hydroxybenzyl)ethylene diamine-N,N′-diacetic acid (HBED) and deferasirox (Def aka DFX) are representative cTfm chelators that possess coordinating moieties comparable to those of the sTf metal binding site. Figure 6a–c highlight in color the comparable binding atoms. For the anticancer strategy, Ti(IV) complexes with the cTfm chelators as ligands were designed to remain intact extracellularly where there is virtually no labile Fe available due to sTf virtually exclusively binding and transporting Fe(III) in blood. Within the intracellular environment, where labile Fe is relatively abundant, the Ti-cTfm complexes were expected to become activated. It was rationalized that via a transmetalation mechanism, the Ti(IV) cTfm complexes would undergo metal exchange with Fe(III) of the LIP to transform Fe(III) into a functionally inert Fe-cTfm species thereby releasing Ti(IV) and enabling its cytotoxic properties [36,37,38,39,40,41,42,43,44,45,46,47].

The structure and formation constants of the Ti-cTfm complexes were examined to evaluate their suitability for the drug design strategy, in particular their stability in aqueous solution and ability to facilitate Fe(III) transmetalation. The Def chelator is an FDA approved tridentate Fe(III) chelator used orally to treat iron overload diseases. It binds Fe(III) in a 1:2 ligand:metal stoichiometry with a high formation constant (log K_Fe(Def)2_ = 38.6) [54]. No X-ray structure has been obtained for the Fe(Def)_2_ complex but a structure has been obtained for the deferasirox analogue 3,5-bis(2-hydroxyphenyl)-1-phenyl-1,2,4-triazole (Figure 6b) [55]. It shows that the two molecules of the ligand coordinate Fe(III) in a meridional arrangement. In aqueous solution from a pH range of 6 to at least 12 and 1:2 metal:ligand ratio and micromolar concentrations, deferasirox is able to maintain Fe(III) stably bound in this 1:2 metal:ligand coordination [55,56]. Def is also able to bind Ti(IV) in this coordination modality as observed in an X-ray structure obtained by Tinoco et al. (Figure 6b) and with a comparable formation constant (log K = 38.8 ± 0.1) [57]. Speciation studies reveal that from a pH of 4 to 8 and 1:2 metal:ligand ratio and micromolar concentrations, the 1:2 metal:ligand complexation is the only species present in an aqueous solution. Despite the comparable formation constants for Def complexation of Fe(III) and Ti(IV), [Ti(Def)_2_]^2−^ is able to effectively transmetalate with a labile source of Fe(III); the citrate complex of Fe(III) ([Fe(citrate)_2_]^5−^) [43]. This is expected to also occur within the intracellular environment given that the influx of Ti(IV) concentration at any given moment is likely not to exceed that of the LIP. The HBED ligand binds Fe(III) and Ti(IV) as a hexadentate ligand with very high affinity to both metal ions but clearly much higher affinity for Fe(III) (log K_FeHBED_ = 39.01 and log K_TiHBED_ = 34.07) [58,59]. An X-ray structure of the [Fe(HBED)]^−^ species was obtained but was of low quality (CCDC 1194788; Ref. [60]). It nonetheless compares favorably with the X-ray structure of the Ti(HBED) neutral compound that was crystallized at pH 3.0 [59]. pH-dependent speciation studies at 1:1 metal:ligand ratio and micromolar concentrations reveal very important differences in HBED coordination of the two metal ions. From a pH of 2.5 to 11, HBED maintains Fe(III) bound in a hexadentate modality. However, above pH 4.0 HBED competes with Ti(IV) induced hydrolysis and transitions from a hexadentate coordination to a pentadentate coordination with one of the phenolate arms becoming dissociated, yielding a titanyl HBED species ([TiO(H^+^-HBED)]^−^). This species was crystallized at pH 7.0 (Figure 6c) [61]. Further hydrolysis occurs above 7.0 further decreasing the denticity of the HBED. Nonetheless, up to at least a pH 10.0, HBED maintains Ti(IV) stably bound in a mononuclear speciation [61]. Like [Ti(Def)_2_]^2−^, [TiO(H^+^-HBED)]^−^ can transmetalate with labile Fe(III) and neither are able to transmetalate with Fe(III)-bound sTf. Both complexes react immediately upon mixture with [Fe(citrate)_2_]^5−^ and reach equilibrium within a timeframe of hours, which is feasible for an intracellular process that could detrimentally affect cell proliferation and survival [41,50,62].

**Figure 7:**
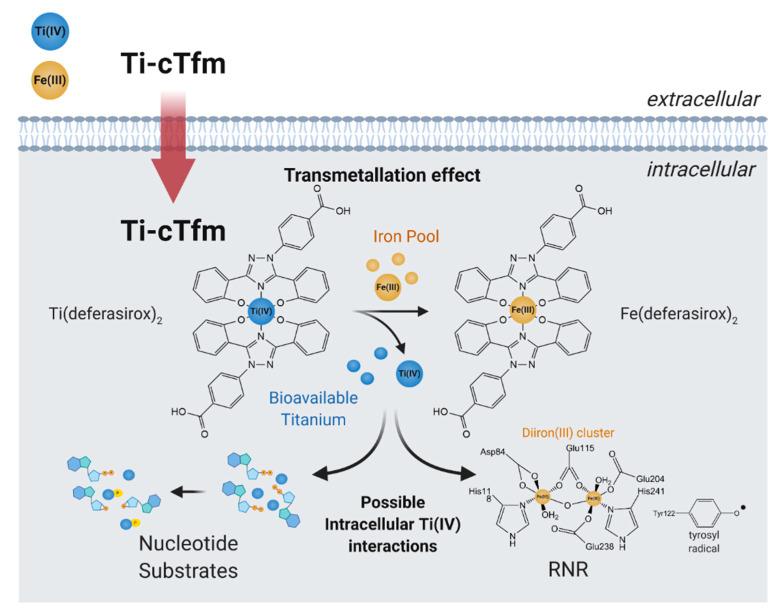
Proposed mechanism of action of the Titanium(IV)-chemical transferrin mimetics (Ti-cTfm).

In cell-based studies, the Ti-cTfm compounds have demonstrated potent antiproliferative and apoptotic properties [43,57,62]. Mechanistic studies reveal the contributions made by the iron chelators and the Ti(IV) ion to this activity (Figure 7). Supplementation of cell lines with Fe improves their viability against treatment with the compounds. The impact that the compounds have on the bioavailability of intracellular Fe has been gauged by monitoring the activity of the RNR enzyme. The enzyme contains an important diiron cofactor site that is responsible for the activation of the enzyme. Fe(II) binding from LIP to this site results in oxidation of the metal ions and the generation of a neighboring tyrosyl radical. This radical is then transported to the active site to initiate the radical-catalyzed reduction of NDPs. The tyrosyl radical can be observed by electron paramagnetic resonance (EPR) at g ~2.0. This radical can be tracked to measure the relative activity of the enzyme. A whole-cell EPR experiment was performed following the EPR signals of Jurkat leukemia cells treated with the Ti-cTfm compounds for a few hours at mid micromolar concentration levels [51,57]. Compared to compound-free controls, the intensity of the tyrosyl radical signal was decreased by up to 75% [57]. The results suggest that the compounds suppress Fe availability and inhibit the activation of the RNR enzyme. In a complementary finding, the Ti(IV) ions display the capacity to not only bind the nucleotide substrates but to also induce phosphate hydrolysis of the substrates [57]. This Ti(IV) activity could prevent RNR recognition of the nucleotide substrates. The ramification of RNR inhibition could be observed in a flow cytometry study that demonstrated that the Ti-cTfm compounds arrest the Jurkat cells at the S phase of the cell cycle, indicative of blocked or decreased DNA replication [57]. Collectively these studies demonstrate the grand potential that the biomimetic Ti-cTfm compounds can have as broad-spectrum anticancer drugs.

## 3. Powder X-ray Diffraction (PXRD) in Drug Development, Formulation, and Polymorphic Characterization

SCXRD has served as an important tool in structural biology for the discovery of new biomimetic APIs and target molecules. It has served to identify the atoms presented in a crystal and their specific locations, along with the electron densities, bond lengths, and angles. For some drugs, obtaining a single crystal is a challenging task and they largely exist in microcrystalline material form. During such instances, PXRD comes to the rescue and provides an alternate tool to establish the structure, purity, efficacy, and effectiveness of a varied range of materials in different disciplines, specifically pharmaceutical science. Unlike SCXRD, which provides the structure within a single crystal, PXRD provides the structural information for a bulk microcrystalline material. The PXRD pattern that is obtained contains the average diffraction for many randomly oriented crystallites in the sample. It is represented as a plot of the diffracted intensities versus the angle of the detector, 2-theta (2-θ). Like SCXRD, the production of a PXRD pattern is defined by Bragg’s Law, according to Equation (1):

(1) $$n\lambda =2d_{hkl}\;sin\;\theta$$

where n is an integer, λ is the wavelength of radiation, $d_{hkl}$ is the interplanar spacing of the set of crystal planes with indices hkl, and θ is the diffraction angle or Bragg angle. Solid forms of a given molecule that exhibit long-range crystalline order can be identified and characterized using PXRD. In company with other techniques, PXRD has served as a successful method in the structural elucidation of several small drug molecules making it important for pharmaceutically relevant materials that can only be produced in the microcrystalline form [63,64,65,66]. More commonly, the PXRD pattern obtained is used to identify whether a polymorphic form of an API is present in a sample of interest. High value pharmaceutical intermediates and APIs may exhibit polymorphism. Polymorphism is the ability of a solid to arrange its atoms in more than one form or crystal structure. The identification of a polymorph is important due to changes in pharmacokinetic properties of one form to another. These changes can affect the ability to process and/or manufacture the drug substance, as well as affect the stability, dissolution, and bioavailability of the drug [67]. Hydrates or solvated crystal forms of an API may also produce distinct pharmacokinetic properties that may lead to an undesirable drug product [67]. More than 90% of pharmaceutical compounds result in more than one solid form or phase [68]. PXRD is the easiest and fastest method to obtain fundamental information about the structure of a material. With a PXRD pattern one can quickly distinguish different phases within a particular material. SCXRD analysis can serve to obtain the three-dimensional structure of new polymorphic forms and can be used for obtaining the calculated PXRD patterns. The experimental PXRD pattern can then be compared to a library database of known or calculated/expected patterns to determine which phase or phases are present within the solid material. Moreover, the inclusion of a PXRD pattern is typically a requirement for new drug candidates, polymorphs, and other potential drug products.

PXRD also serves to confirm the crystalline or amorphous nature of a compound of interest. In crystalline material, there is a periodic arrangement of atoms in three-dimensional (3-D) space. X-rays are scattered only in specific directions when they strike the formed framework, causing the well-defined high intensity Bragg peaks. Amorphous materials contain atoms that do not possess periodicity and are ordered primarily at short-range order (2–5 Å) and medium-range order (5–20 Å) distances [69]. The X-rays are scattered in many directions leading to a large bump distributed in a wide range 2-θθ angle [70]. For many APIs the amorphous form exhibits significant advantages over the crystalline form including higher solubility, dissolution rate, and oral bioavailability [71,72]. This advantage has led to the development and approval of several amorphous APIs with the desired pharmacokinetic properties. However, due to the higher free energy of some amorphous systems, they tend to be thermodynamically unstable and therefore lead to crystallization of the API, especially if exposed to high humidity [71]. Hence, PXRD has served to identify the onset of crystallization in amorphous APIs and can set a detection limit for the crystalline content in drug formulations, typically on the order of a few percent (0.2–5%) crystallinity by mass and lower with the use of synchrotron X-ray sources [73,74].

## 4. The Pair Distribution Function (PDF)

Of late, the application of PXRD to study amorphous APIs has permitted direct characterization of amorphous construction by the PDF. The PDF (*G(r)*) is attained by performing Fourier transformation of the total scattering structure function designated as *S(Q)*, as shown in Equation (2):

(2) $$G\left(r\right)=\frac{2}{\pi }\int_{Q=0}^{Q_{max}}Q\left[S\left(Q\right)-1\right]sinQrdQ$$

where *Q* is the magnitude of the wave vector (*Q* = 4*π* sin*θ*/*λ*), 2*θ* is the scattering angle and λ is the wavelength of the incident X-ray beam [75]. *S*(*Q*) can be related to the total coherent scattered X-ray intensities, by Equation (3):

(3) $$S\left(Q\right)=1+\frac{I^{coh}\left(Q\right)-\sum ic_{i}f_{i}{\left(Q\right)}^{2}}{\sum c_{i}f_{i}{\left(Q\right)}^{2}}$$

where *I^coh^* (*Q*) is the measured scattering intensity obtained from the sample. Here *I^coh^* (*Q*) is adjusted for background and other experimental properties and normalized by the X-ray incident flux and the number of atoms in the sample; *c_i_* is the atomic concentration and *f_i_* the X-ray scattering factor for the atom of type *i*.

The PDF generates the probability to detect an atom at an estimated distance (*r*) from any reference atom. As such, PDF postulates data on local structure in real space. The *r* expressed in the PDF data is a representation of the peaks generated by intermolecular and intramolecular interactions, considering their abundance in the molecular system. Sharp intramolecular peaks at small distances are representative of robust covalent bonding inside a molecular system. The initial few peaks often characterize carbon-carbon nearest-neighbor or next nearest-neighbor bonds. Broader intermolecular peaks at farther distances characterize the alignment and arrangement between molecules. PDF can measure the atomic structure of a material in the magnitude of 1 to 100s of an angstrom (Å) with the use of laboratory and high energy light sources hence allowing for a direct measurement of the local structure at short and medium range order [76,77]. A more detailed idea of the principles and the general application of PDF in PXRD is presented by Egami and Billinge [78].

### 4.1. The PDF in Drug Polymorphism

**Figure 8:**
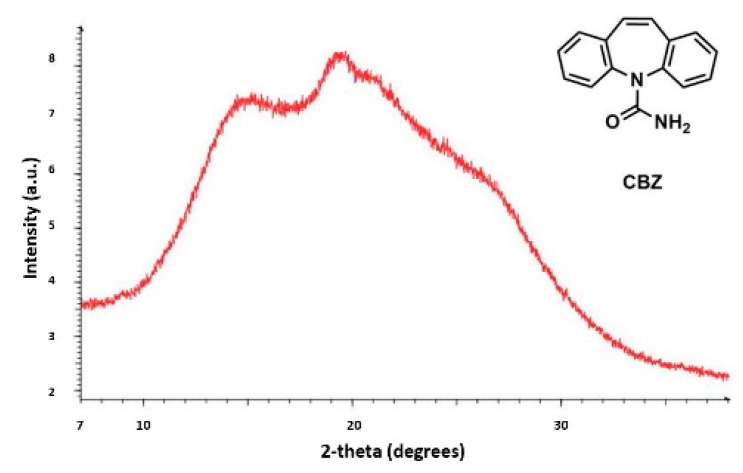
Powder X-ray diffraction (PXRD) patterns from a conventional X-ray source of the amorphous active pharmaceutical ingredient (API) of carbamazepine (CBZ) prepared by melt. Republished with permission of Royal Society of Chemistry, from Characterisation of amorphous and nanocrystalline molecular materials by total scattering, Billinge, S.J.L.; Dykhne, T.; Juhás, P.; Božin, E.; Taylor, R.; Florence, A.J.; Shankland, K. 12, 2010; permission conveyed through copyright clearance center, Inc. *Crystal Engineering Communications* **2010**, *12*, 1366–1368, doi:10.1039/b915453a. The enhanced version of the figure was obtained from the thesis of Davis, T.D., Fingerprinting analysis of non-crystalline pharmaceutical compounds using high energy X-rays and the total scattering pair distribution function, in Department of Applied Physics and Applied Mathematics. 2011, Columbia University [82].

**Figure 9:**
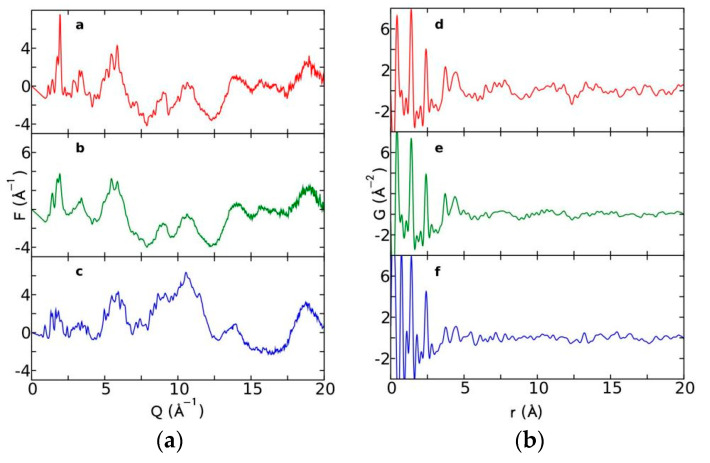
(**a**) Total scattering diffraction patterns and (**b**) PDFs of the various carbamazepine (CBZ) samples; a and d correspond to crystalline CBZ form III, b and e correspond to the amorphous CBZ prepared by melt and c and f correspond to crystalline CBZ form I. Republished with permission of Royal Society of Chemistry, from Characterisation of amorphous and nanocrystalline molecular materials by total scattering, Billinge, S.J.L.; Dykhne, T.; Juhás, P.; Božin, E.; Taylor, R.; Florence, A.J.; Shankland, K. 12, 2010; permission conveyed through copyright clearance center, Inc. *Crystal Engineering Communications* **2010**, *12*, 1366–1368, doi:10.1039/b915453a. The enhanced version of the figure was obtained from the thesis of Davis, T.D., Fingerprinting analysis of non-crystalline pharmaceutical compounds using high energy X-rays and the total scattering pair distribution function, in Department of Applied Physics and Applied Mathematics. 2011, Columbia University [82].

**Figure 10:**
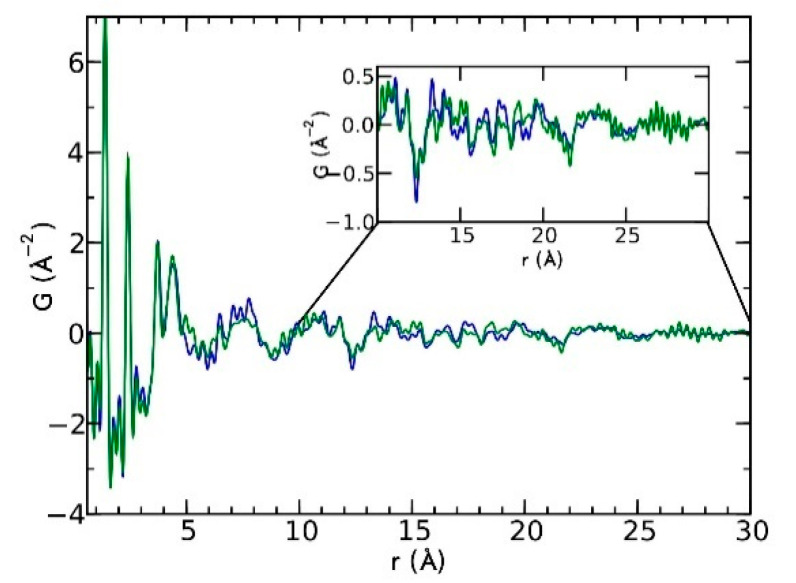
Comparison of the PDF data from the amorphous carbamazepine (CBZ) prepared by melt (green) and crystalline CBZ form III (blue). Republished with permission of Royal Society of Chemistry, from Characterisation of amorphous and nanocrystalline molecular materials by total scattering, Billinge, S.J.L.; Dykhne, T.; Juhás, P.; Božin, E.; Taylor, R.; Florence, A.J.; Shankland, K. 12, 2010; permission conveyed through copyright clearance center, Inc. *Crystal Engineering Communications* **2010**, *12*, 1366–1368, doi:10.1039/b915453a. The enhanced version of the figure was obtained from the thesis of Davis, T.D., Fingerprinting analysis of non-crystalline pharmaceutical compounds using high energy X-rays and the total scattering pair distribution function, in Department of Applied Physics and Applied Mathematics. 2011, Columbia University [82].

Currently, the identification of polymorphic forms in nanocrystalline and amorphous APIs through conventional crystallography methods is difficult due to the diffraction features becoming too broad and not useful for differentiating between local molecular packing arrangements. Figure 8 shows the molecular structures of carbamazepine (CBZ) and the X-ray diffraction pattern of the amorphous API formulated by the melt technique and characterized by a conventional X-ray source. The PXRD diffractogram is broad and not useful for differentiating between local molecular packing arrangements. Nonetheless, the PDF can be used to obtain molecular insight into API polymorphism. Carbamazepine (CBZ), sold under the commercial name Tegretol, is an anticonvulsant and mood alleviating medication used mainly for the treatment of epilepsy and is known to exist in five polymorphic forms and an amorphous form [79]. Of the polymorphs, form III is the commercially available form and the most stable at room temperature. CBZ is a Class 2 drug according to the BCS. Absorption of the APIs is limited by its solubility. Billinge et al. used a high energy synchrotron source with a λ = 0.137 Å to obtain the total scattering atomic PDF to study the amorphous forms of CBZ [80]. The *G(r)* was obtained from the total scattering structure function, using the program PDFgetX2 [81]. The short wavelength used allowed the data collection over a higher *Q*-range providing an ideal resolution in real-space for quantifiable structural analysis. Figure 9 shows the overall scattering data, presented as *F(Q)*, and the PDFs data, presented as *G(r)* for both the amorphous and crystalline samples of CBZ. The *F(Q)* distinguishes differences in the crystalline samples of CBZ form III and form I (Figure 9 samples a and c respectively). The amorphous CBZ sample prepared by the melting technique shows similarity to that of CBZ form III in both total scattering data and PDF data. When overlaying the PDF data of CBZ form III and the amorphous CBZ prepared by melt (Figure 10) there is evidence that the local packing in the API formed by the melt is identical to that of CBZ form III. It was concluded that the sample prepared by melt of CBZ formed nanocrystalline domains of CBZ form III. In this work, PDF allowed the detection of small variations in the nanocrystalline materials that can be used to identify and distinguish different known forms of the crystalline polymorphs.

### 4.2. Pair Distribution Function (PDF) in Amorphous Solid Dispersions (ASD) Formulations

To help improve the stability of amorphous APIs, they can be formulated with an excipient to aid in maintaining their amorphous state [83,84]. These types of formulations are called amorphous solid dispersions (ASDs) and are produced by a mixture of the amorphous form of an API and a second component, typically a polymer. These mixtures are known to improve the stability of the bioactive compound and can contribute to their enhanced dissolution rate and absorption properties, increasing the overall bioavailability of the APIs [85]. A variety of polymers are used as excipients in ASDs. Polymers are chemically constituted of repeated structural units known as monomers which are associated together to form an extended structural framework or matrix [86]. The polymer matrix allows for the incorporation of the amorphous API. The polymers consist of those that are natural (starch, silk, wool, cellulose, DNA and proteins), semisynthetic (nitrocellulose, hydroxypropyl methylcellulose [HPMC]), or synthetic (polyvinylpyrrolidone [PVP]) [87]. Polymers can also be amorphous (polyvinyl chloride (PVC), polyacrylic acid [PAA]), semi-crystalline (poly l-lactic acid [PLLA]), or crystalline (polyethylene glycol [PEG]). Various studies have shown that ASDs containing excipients derived from natural sources exhibit enhanced solubility and dissolution properties [88,89]. These biomimetic drug formulations are highly sought after as they have many benefits including lack of toxicity, availability, lower costs in manufacturing, biocompatibility, and biodegradability [90,91]. Enhanced drug functionality can be obtained from bioactive excipients [90,91]. The Handbook of Pharmaceutical Excipients contains a list of excipients that can be used in pharmaceutical drug products and typically used to develop ASDs [92]. Both Ogaji et al. and Saha et al. provide a list of natural excipients that can also be used to develop ASDs [93]. Table 1 displays a limited list of different polymers used to formulate some of the commercially available ASDs along with their glass transition temperature (T_g_), an important factor for describing the physical properties of polymers [94].

ASDs are produced using techniques such as fast evaporation from solvent (rotary evaporator, spray-drying, and dry-freeze) and melt techniques [83,95,96,97]. The most commonly used methods are spray drying (SD) and hot-melt extrusion (HME) [98,99]. SD is a solvent evaporation method that converts solutions to powders in a single process. The procedure consists of dissolving the drug and polymer in an acceptable pharmaceutical Class 3 (low toxicity) solvent according to Pharmaceuticals for Human Use (ICH) guidance for industry Q3C and evaporating the solvent into very fine particles. Because the process is extremely fast it can be used to prepare ASDs with compounds having poor thermal stability. HME is the preferred method in pharmaceutical development because it is a one-step process that contains no solvents, making the process cost effective and less prone to toxicological and environmental hazards. The process consists of employing heat and pressure to soften a polymer and force it past an opening in an uninterrupted process.

ASDs can be organized into amorphous one-phase systems, amorphous two-phase systems, and crystalline-amorphous two-phase systems [100]. The most favorable drug-polymer mixture is a one phase system. In this system all molecules of the amorphous API are homogeneously mixed with the polymer molecules. The resulting mixture can contain interactions between the amorphous API and polymer that are stronger than the interactions between the components alone [101]. These interactions include hydrogen bonding, van der Waals forces, electrostatic, ionic, and hydrophobic forces [95]. These interactions can restrict the molecular mobility of the APIs and delay or prevent nucleation and crystal growth (crystallization). Factors that affect the physical stability of the drug products are classified into thermodynamic, kinetic, and environmental aspects. The formation of a stable one phase system is mainly dependent on the thermodynamic miscibility and solubility of the drug and polymer [102]. The T_g_, molecular mobility, manufacturing process, physical stability of the amorphous API, and drug-polymer interactions are considered as the kinetic factors which are associated with the stability of ASDs [103,104,105]. Storage and environmental conditions including temperature and humidity can also affect the stability of ASDs. A change in conditions can cause phase separation of the homogenous one phase system, increasing the risks of API crystallization and converting it into a two-phase system [106,107,108].

Initially, PXRD has served to confirm the absence of crystalline phases in ASDs [109,110]. The crystallization of the API in ASDs because of crystalline-amorphous phase separation or instability can be easily detected given the exceedingly well-organized nature of crystalline materials. However, the use of PXRD to probe miscibility or to detect amorphous-amorphous phase separation is much more difficult due to the lack of long-range ordering of molecules in ASDs. The PDF can be used to provide a direct measurement of the local structure in ASDs. Changes in nearest-neighbor interactions due to drug-polymer interactions can be detected with this method. PDF analysis can differentiate between a completely miscible and partially miscible system. Initially, Nollenberger et al. applied PDF to explore the molecular structure of ASDs containing the API felodipine and compared the data obtained to dissolution studies [111]. It was observed that the dissolution behavior was influenced by particle characteristics such as the size and specific surface area of the ASDs and also non-covalent interactions between the API and the polymer. In contrast to standard methods of evaluation (differential scanning calorimetry, PXRD, infrared spectroscopy, Raman, ^13^C-NMR), PDF analysis was able to describe the local ordering and identify variances among the various ASDs. Here, the first studies using PDF, obtained from a conventional copper (Cu) K-α X-ray laboratory source, could be used to study ASDs mixtures. However, limitations to the use of conventional sources including high noise levels or low resolution may have led to an unclear conclusion in this specific study [112].

**Figure 11:**
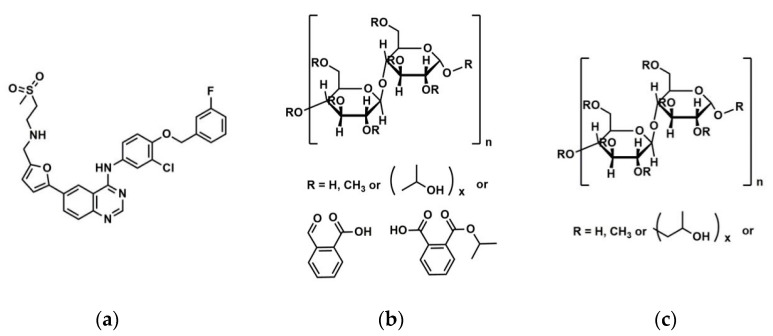
Molecular structures of (**a**) lapatinib (LP) (**b**) hypromellose phthalate (HPMCP) and (**c**) hypromellose (HPMC-E3).

**Figure 12:**
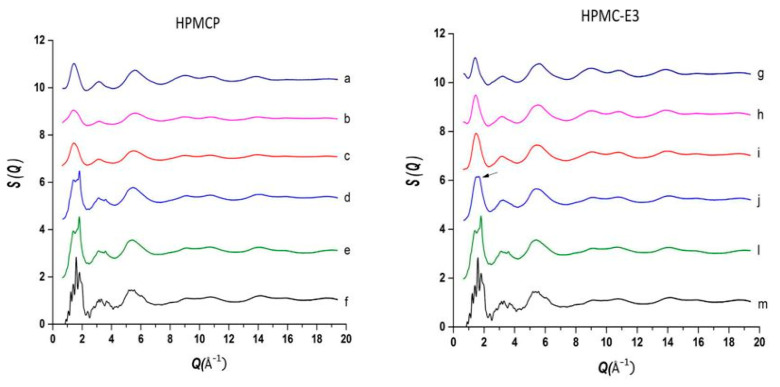
Comparison of the measurement of total scattering structure factors for spray dried (SD) lapatinib (LP) free-base, polymers and their mixtures: (**a**) pure hypromellose phthalate (HPMCP) (SD); (**b**) 1:3 LP:HPMCP; (**c**) 1:1 LP:HPMCP; (**d**) 3:1 LP:HPMCP; (**e**,**l**) pure amorphous LP (SD); (**f**,**m**) crystalline lapatinib raw material (as is, not spray dried); (**g**) pure Hypromellose (HPMC-E3) spray dried; (**h**) 1:3 LP:HPMC-E3; (**i**) 1:1 LP:HPMC-E3; (**j**) 3:1 LP:HPMC-E3, arrow indicates residual crystallinity that was detected. The figure was taken from de Araujo, G.L., C.J. Benmore, and S.R. Byrn, Local Structure of Ion Pair Interaction in Lapatinib Amorphous Dispersions characterized by Synchrotron X-Ray diffraction and Pair Distribution Function Analysis. Scientific Reports, 2017. 7: p. 46367. Copyright (2017), with permission from Springer Nature [113].

The use of high energy light sources such as synchrotron sources in combination with PDF has provided more reliable data when compared to conventional X-ray instruments. The use of high-energy X-rays with shorter wavelengths can allow access to the structural information present at higher *Q* ranges, where *Q = 4π sinθ/λ,* thus allowing a higher detection range to be achieved. Araujo et al. utilized synchrotron X-ray diffraction and PDF to explore the local chemical scaffold and ionic drug-polymer interactions in ASDs comprising of lapatinib (LP) as the API and the biomimetic excipients hypromellose phthalate (HPMCP) and hypromellose (HPMC-E3) (Figure 11) [113]. HPMC is produced from cellulose, a natural polymer and fiber, which is considered to be safe for human consumption. HPMCP is a phthalic acid ester of hydroxypropyl methylcellulose. It serves the additional purpose as an enteric coating to protect drugs against degradation by gastric acid when orally delivered. The ASDs investigated were formulated by SD at drug:polymer ratios of 3:1, 1:1 and 1:3. LP is categorized as a BCS Class II API and is currently used in breast cancer therapy [114]. The X-ray diffractograms from a conventional Cu Kα radiation (λ = 1.5418 Å) X-ray source were obtained. The data confirmed that all ASD mixtures were amorphous and presented no differences in the shape of the amorphous peaks. To assess the structural information at higher *Q*-ranges the mixtures were analyzed by a high energy synchrotron source. The high-energy X-ray experiments were performed using a monochromatic beam of energy radiation equivalent to 100.315 keV (λ = 0.12360 Å) and the total X-ray structure factors S(*Q*) were extracted using PDFgetX2. Figure 12 shows the contrast of measured total X-ray structure factors obtained from the synchrotron source for all the LP-HPMCP and LP-HPMC-E3 mixtures and pure API. The X-ray pattern from the synchrotron source detected differences in the mixtures that the conventional X-ray source did not, allowing detection levels as low as 0.2% (*w*/*w*) of crystallinity to be reached. The X-ray structure factors of the ASDs prepared at ratios of 1:3 and 1:1 did not present any Bragg peaks, and Araujo et al. assumed the amount of polymer over 50% produced stable ASDs. LP-HPMCP produced at the ratio of 3:1 showed a structure factor very similar to the pure LP which was indicative of residual crystalline API. LP-HPMC-E3 also produced at 3:1 shows a slight deformation in the peak at approximately *Q* = 1.7 Å^−1^ suggesting that the sample is predominantly amorphous but may also present trace amounts of crystalline API. To confirm the stability of the 3:1 mixtures, the ASDs were analyzed 100 days later which showed that the mixtures recrystallized into the more stable crystalline LP form. This indicated that the samples contained enough trace amounts of crystalline API to promote complete recrystallization of the ASDs.

Interactions between the bioactive molecules and polymer matrix play an important role in drug formulations. Strong API polymer interactions including ionic interactions and hydrogen bonds help to improve the physical stability of the drug product and contributes to the dissolution of the bioactive molecule. The API:polymer ratio can also contribute to the stability due to the amount of interactions in the system. Lapatinib (LP) is a weak base comprising of a secondary amine group (pK_a_ = 7.26) and can be mixed with acidic polymers to form strong interactions [115]. A high-energy synchrotron source was used to obtain the PDF data from the intermolecular structure factors of the ASD mixtures to better understand the API:polymer interactions. The intra- and intermolecular structure factors ($S_{LP-LP}^{intra}\left(Q\right)$ and $S_{LP-LP}^{inter}\left(Q\right)$) for the API were obtained from the X-ray intermolecular scattering factor (XISF) method described by Mou et al. [116]. The $S_{polymer-polymer}^{intra}\left(Q\right)$ and $S_{polymer-polymer}^{inter}\left(Q\right)$ for the polymer were obtained from the pure polymer structure factor. The intramolecular structure factors were then subtracted lo leave the API-API and API-polymer intermolecular interactions alone ($S_{LP-LP}^{inter}\left(Q\right)$ and $S_{LP-polymer}^{inter}\left(Q\right)$). With the application of a Fourier transform the intermolecular S(*Q*), in reciprocal space, could be converted into the differential PDF, *D(r)* [117], as shown in Equation (4):

(4) $$D\left(r\right)=\frac{2}{\pi }\int_{Q=0}^{Q_{max}}Q\left[S\left(Q\right)-1\right]sinQrdQ$$

Similar to the *G*(*r*), the *D(r)* characterizes the probability of finding an atom of radial distance (*r*) on either the API or polymer surrounding an atom on an API molecule at the origin and can be expressed in terms of the total differential PDFs for the API-API and API-polymer intermolecular interactions, as Equation (5):

(5) $$\Delta D_{API}^{inter}\left(r\right)=\;D_{API-API}^{inter}\left(r\right)+D_{API-polymer}^{inter}\left(r\right)$$

**Figure 13:**
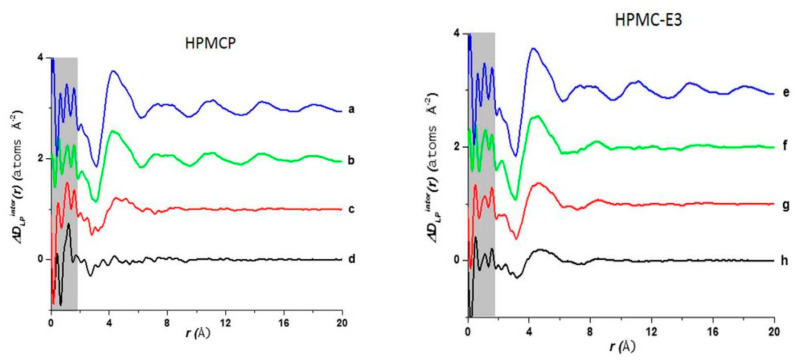
Comparison of the intermolecular differential pair distribution functions of drug-polymer mixtures: (**a**,**e**) pure lapatinib (LP) (spray dried (SD)); (**b**) 3:1 LP:HPMCP; (**c**) 1:1 LP:HPMCP; (**d**) 1:3 LP:HPMCP; (**f**) 3:1 LP:HPMC-E3; (**g**) 1:1 LP:HPMC-E3; (**h**) 1:3 LP:HPMC-E3. The gray area represents the low-r density region where systematic errors are more prone. The figure was taken from de Araujo, G.L., C.J. Benmore, and S.R. Byrn, Local Structure of Ion Pair Interaction in Lapatinib Amorphous Dispersions characterized by Synchrotron X-Ray diffraction and Pair Distribution Function Analysis. Scientific Reports, 2017. 7: p. 46367. Copyright (2017), with permission from Springer Nature [113].

Figure 13 shows a comparison of *D(r)* of the drug-polymer mixtures and the amorphous API alone. The amorphous PDF pattern for the API alone shows well-defined atom to atom interactions reaching out to *r* = 20 Å. The ASDs produced from LP:HPMCP at a ratio of 3:1 display a similar pattern to the API indicating that the sample is drug rich. The 1:1 mixture shows a broad peak at approximately 4.3 Å signifying that the presence of a repetitive pattern only extends out to the nearest neighbor atom. The 1:3 mixture displays a flat line indicating that the API is most likely homogeneously distributed in the polymer making this concentration the most stable. In the case of the LP:HPCM-E3 mixtures the drug rich 3:1 composition shows a different shift at ~4.4 Å and a very weak second peak at ~8.3 Å and nothing further at higher *r*. Both the mixtures at ratios of 1:1 and 1:3 show similar patterns to that of the 3:1 mixture. The first and second peaks are present but are reduced at higher magnitudes owing to the small drug concentration. Therefore, it is seen that LP molecules, at elevated concentrations in the LP-HPMC-E3 mixtures, did not arrange in a similar way as observed in LP-HPCMP mixtures. LP is well identified to have ionic interactions with HPMCP via an acid-base mechanism that can be undoubtedly detected by the alterations in color of the final dispersions. The LP-HPMC-E3 dispersions exhibit a pale-yellow color and the LP-HPMCP samples show a bright yellow color. This interaction was not anticipated in LP-HPMC-E3 mixtures because of the absence of carboxylic groups that can react with amines from the API. When there is an acid-base interaction, the native structure is interrupted causing the drug molecules to be disrupted by the acidic functional group present on the polymer, thereby enhancing the disorder of the entire system and rendering its bright color. It was concluded that the absence of peaks in the PDF pattern of the 1:3 LP:HPMCP mixture suggested that a thorough reaction occurred with the polymer. These studies indicate that 1:3 LP:HPMCP is more stable than 1:1 LP:HPMCP and 1:3 LP:HPMC-E3. To confirm this finding, a stress experiment was performed which revealed that both samples started to crystallize after 7 days for the 1:1 LP:HPMCP dispersions and 30 days for the 1:3 LP:HPMC-E3 dispersions, while 1:3 LP:HPMCP dispersion remained amorphous. This finding validates the use of ASDs to stabilize drug formulations and the use of PDF to confirm miscibility.

## 5. Alternative Approaches/Techniques Available for Structure Determination in Drug Discovery

Although X-ray diffraction techniques play a pivotal role in drug discovery to study the structural biology, polymorphism, and formulation of the drug and to validate the drug-target protein/biomolecular interactions, there are other well established complementary methods that are utilized especially in cases where it is not possible to obtain crystal structures. Some such techniques include Paramagnetic Solution Nuclear Magnetic Resonance (NMR) Spectroscopy [118,119,120,121,122], Solid State NMR, Electron cryo-Microscopy (cryo-EM) [123,124], and Computational Analysis [125,126,127,128,129,130]. Discussion of these techniques goes beyond the scope of this review article but we provide citations for several important review articles discussing the application of these techniques for drug discovery.

## 6. Conclusions

X-ray structural characterization techniques play significant roles in drug discovery, design, and formulation. Accurate analysis of crystal structures of biological target and biological target-substrate complexes is crucial at all stages. However, these models have their limitations. Crystal structures can be over analyzed and this could lead to biased hypotheses and unlimited series of downstream experiments. Thus, critical evaluation of small molecule and macromolecular structures and their physiologically relevant interactions with the use of complementary techniques is required before taking conclusive decisions in drug discovery.

The PDF is a promising analytical approach that allows the study of both crystalline and amorphous materials. The experiments are non-destructive and the use of the short-wavelength X-rays helps to elucidate the inner structure of a solid material. PDF provides information regarding the molecular ordering down to the atomic and nm level. Differences in the molecular local ordering of amorphous APIs and drug compounds such as ASDs can now be detected using PDF. This technique can be used to determine intermolecular interactions between the drug and polymer carriers of ASDs and can provide a fingerprint of amorphous APIs. It can also be used to identify structural trends that will help predict macroscopic behaviors in amorphous drug products.

Additional X-ray based techniques can be used in drug discovery and development for the structural characterization of drug compounds such as small-angle X-ray scattering (SAXS) and wide-angle X-ray scattering (WAXS). These techniques can be useful to study drug delivery systems and large biological components such as proteins and can also be used to study crystallization processes of drugs from solution. Here we focused on X-ray crystallography techniques such as SCXRD and PXRD complimented with PDF to determine the molecular arrangement of biomimetic compounds and to distinguish solid forms in drug formulations.

## Figures and Tables

**Table 1:** Overview of commercially available amorphous solid dispersions (ASDs), their manufacturer, active pharmaceutical ingredient (API), the polymer used to produce the ASDs, and the glass transition temperature (T_g_) in Celsius (°C) of the polymer. Ref. [86,94].

ASD Name	Manufacturer	API	Polymer	T_g_ of Polymer (°C)
Gris-PEG^®^	Pedinol Pharmacal INC	Griseofulvine	PEG6000	~60
Cesamet^®^	Valeant Pharmaceuticals	Nabilone	PVP	130–160
Kaletra^®^	Abbott	Lopinavir, Ritonavir	PVPVA	106
Sporanox^®^	Janssen Pharmaceutica	Itraconazole	HPMC	172
Intelence^®^	Tibotec	Etravirine	HPMC	172
Certican^®^	Novartis	Everolimus	HPMC	172
Isoptin^®^ SR-E	Abbott	Verapamil	HPC/HPMC	143–172
Nivadil^®^	Fujisawa Pharmaceutical Co., Ltd.	Nivaldipine	HPMC	172
Prograf^®^	Fujisawa Pharmaceutical Co., Ltd.	Tcrolimus	HPMC	172
Rezulin^®^	Developed by Sankyo, manufactured by Parke-Davis division of Warner-Lambert	Troglitazone	PVP	130–160

## Data Availability

Publicly available datasets were analyzed in this study. This data can be found here: The Cambridge Crystallographic Data Centre and the RCSB Protein Data Bank.
